# Biologic Therapies for Asthma and Allergic Disease: Past, Present, and Future

**DOI:** 10.3390/ph16020270

**Published:** 2023-02-10

**Authors:** Fernando Ramírez-Jiménez, Gandhi Fernando Pavón-Romero, Juancarlos Manuel Velásquez-Rodríguez, Mariana Itzel López-Garza, José Fernando Lazarini-Ruiz, Katia Vanessa Gutiérrez-Quiroz, Luis M. Teran

**Affiliations:** Immunogenetics and Allergy Department, Instituto Nacional de Enfermedades Respiratorias Ismael Cosio Villegas, (INER), Mexico City 14080, Mexico

**Keywords:** asthma, biologic therapies, monoclonal antibodies, cytokines, chemokines

## Abstract

The discovery of the mechanism underlying allergic disease, mouse models of asthma, and bronchoscopy studies provided initial insights into the role of Th2-type cytokines, including interlukin (IL)-4, IL-5 and IL-13, which became the target of monoclonal antibody therapy. Omalizumab, Benralizumab, Mepolizumab, Reslizumab, and Tezepelumab have been approved. These biologicals have been shown to be good alternative therapies to corticosteroids, particularly in severe asthma management, where they can improve the quality of life of many patients. Given the success in asthma, these drugs have been used in other diseases with type 2 inflammation, including chronic rhinosinusitis with nasal polyps (CRSwNP), atopic dermatitis, and chronic urticaria. Like the Th2-type cytokines, chemokines have also been the target of novel monoclonal therapies. However, they have not proved successful to date. In this review, targeted therapy is addressed from its inception to future applications in allergic diseases.

## 1. Introduction

The discovery of murine T helper 1 (Th1) and T helper 2 (Th2) cell clones has had a high impact on the development of novel therapeutic targets for allergy disease. In 1986 Mossman and Coffman reported that Th1 clones produced interferon (IFN)-y while Th2 clones released IL-4 [1]. Soon after, it was demonstrated that Th2 clones produced IL-5, IL-6, and IL-10 [2]. Th1 clones were involved in helper activity for cell-mediated immunity, while Th2 clones stimulated B-cell development and antibody production. In parallel, the nomenclature of type-2 (T2) cytokines began to be used to describe the immunologic effect of these cytokines [2]. Currently, type-1 cytokines are IFN-y, IL-2, and IL-12 while type-2 cytokines include IL-4, IL-5, IL-6, IL-10, and IL-13. By undertaking bronchoscopy studies, other researchers and we demonstrated that type 2 cytokines are produced in the airways of allergic asthma patients [3,4,5]. More recently, two specific endotypes, including type 2 high- and low asthma, were described in both allergic and nonallergic eosinophilic asthma, respectively. Allergic asthma involves the production of cytokines such as IL-4, IL-5, and IL-13 produced by the classic Th2 cells, whereas in nonallergic asthma, the airway epithelium release mediators such as thymic stromal lymphopoietin (TSLP) IL-25, and IL-33 upon activation of pollutants or allergens; these cytokines, in turn, activate innate lymphoid cells–type 2 (ILC-2) to release IL-4, IL-5, and IL-13 [6].

The first report of successful therapy with murine monoclonal antibodies in humans was in 1982 in a patient with lymphoma [7], and the first therapeutical murine monoclonal antibody was approved in 1986 for the prevention of kidney transplant rejection. However, it was not until the late 1990s that chimeric monoclonal antibodies were approved and marketed [8]. The development of mAbs has played an important role in the therapy of different disorders, with no exception in the treatment of immune allergic diseases, mainly asthma and skin diseases such as chronic urticaria and atopic dermatitis. Therapeutic mAbs are typically immunoglobulin (Ig) G isotypes; they can be murine (suffix: -omab), chimeric (suffix: -ximab), humanized (suffix: -zumab), or human (suffix -umab) [9].

Asthma has been one of the main objectives in developing mAbs for immunoallergic respiratory diseases; however, the pathophysiology shared between this and other diseases have allowed its use in other conditions, with indications approved by consistent trials. Asthma is a chronic airway disease. It is characterized by variable expiratory airflow limitation and clinically represented by cough, wheezing, shortness of breath, and chest tightness. Asthma is a heterogeneous condition in both children and adults; scientific advances in recent decades have made it possible to understand this heterogeneity, describe disease pathogenesis, and develop new therapeutic strategies, especially in severe diseases [6]. An important molecular mechanism of asthma is type 2 inflammation, which occurs in many but not all patients. The cells involved in type 2 immune response of the airway are Th2 cells, IgE-producing B cells, ILC2s, mast cells, basophils, and eosinophils. This type of inflammation is characterized by the production of interleukin (IL)-4, IL-5, and IL-13, which are produced and released by cells of the adaptive and innate response, such as Th2 and ILC2s, respectively; ILC2 are activated by epithelial damage through the release of TSLP, IL-25, and IL-33 [10].

Soon after the demonstration of the role of Th1 and Th2 cells, a novel family of chemotactic cytokines began to be unraveled, which was named “the chemokines.” These cytokines have been subdivided into four subfamilies based on the position of either one or two cysteine residues located near the N-terminus of the protein: CXC, CC, C, and CXXXC. subfamilies that exert their activity through specific chemokine receptors. Among these chemokines, the CCL family attracted [11] the most attention as it exerted potent quimiotactic activity for eosinophils and lymphocytes. Using the endobronchial allergen challenge, we demonstrated that eosinophil-activating CCL chemokines MIP-1α (CCL3), RANTES (CCL5), and MCP-3 (CCL7) were released into the airways of asthmatic patients; levels of these cytokines peaked at 4 h and declined at 24 h [12]. Subsequently, Brown et al. showed that eotaxin-1 (CCL11) also has a similar release; levels of CCL11 peaked at four hours and declined at 24 h after the allergen challenge [13]. Interestingly, our model of asthma using endobronchial challenges demonstrated that IL-5 shows different kinetics of release compared with chemokines [3]. Levels of IL-5 increase gradually in BAL fluid, which peaks 24 h after the challenge, suggesting that IL-5 may maintain lung eosinophilia whereas chemokines may exert biological activity for a shorter time (4-fold lesser length of time). Our IL-5 study was undertaken up to 24 h following an allergen challenge, and it is not known whether IL-5 may be produced locally for a longer period of time.

Chemokines exert their activities by binding chemokine receptors which belong to the G-protein-coupled receptors (GPCRs). These receptors have become one of the most successful therapeutic targets, and they account for approximately one-third of all Food and Drug Administration (FDA)-approved pharmaceutical drugs. Thus, chemokine receptors are promising drug targets in allergy [14]. The chemokine receptor (CCR)3 attracted the most attention since the discovery of chemokines as it is expressed on eosinophils and Th2 lymphocytes and serves as a receptor for CCL5, CCL11, CCL24, and CCL26 [11,15]. Indeed, CCR3-null mice and eotaxin-1 and eotaxin-2 double-knockout mice abolished up to ~99% of allergen-induced airway eosinophilia [15]. Chemokine receptors expressed on Th2 lymphocytes have also been investigated, including CCR4 and CCR8 [14]. CCL17 (TARC) and CCL22 (MDC) are ligands for CCR4, while CCL1 (I-309) binds CCR8, and these three ligands have been associated with asthma [16,17,18]. The pharmaceutical industry has developed antagonists which also target CCR3, CCR4, and CCR8. For example, GSK developed the molecules GW824575 and GSK2239633, which target CCR3 and CCR4, respectively. However, they did show efficacy in asthma [19]. To date three drugs targeting chemokine receptors have been approved: an anti-CXCR4 antagonist that mobilizes hematopoietic stem cells and is used in oncology (plerixafor), a CCR5 antagonist used for HIV treatment (maraviroc), and a monoclonal-antibody CCR4 antagonist for the treatment of mycosis fungoides or Sézary syndrome (mogamulizumab) [20]. The novel CCR3 antagonist, R321, has been developed, and it is currently under investigation. R321 inhibits G-protein-mediated processes and promotes the endocytosis and degradation of CCR3 [21].

**Figure 1:**
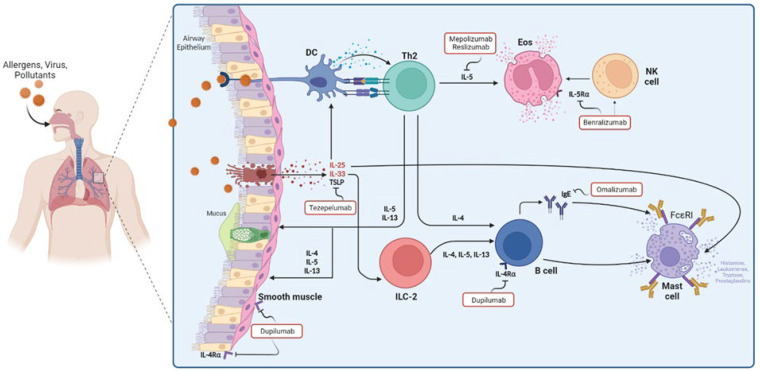
Cells, cytokines, and mediators involved in asthma pathophysiology and the mechanisms of targeted therapy that have been approved in asthma.

Targeted therapy through mAbs has been focused on mitigating the biological effect of substances produced in type 2 inflammatory response. Here, novel anti-cytokine therapies for asthma and allergy disease are reviewed (Figure 1).

## 2. Omalizumab

Omalizumab (Xolair^®^, before called rhuMab-E25) is a recombinant humanized monoclonal antibody that binds directly to the Fc portion of the circulating IgE, preventing the activation of mast cells and basophils, and the release of histamine and tryptase, responsible for the clinical symptoms of atopic disease [22,23]. Moreover, by reducing serum-free IgE, Omalizumab downregulates FcεRI expression on mast cells, basophils, dendritic cells, and IgE-CD23 [24]. Omalizumab was first tested in vitro models as well as animal models [25,26]. At the end of the 20th century, the first randomized, double-blind, placebo-controlled clinical trials were carried out in the United States. They showed that Omalizumab decreased corticosteroid consumption compared to placebo. However, statistically significant results were not obtained [27].

Omalizumab for asthma and allergic rhinitis. The first double-blind placebo-controlled randomized clinical trial in asthma was published in 1997. The response to Omalizumab treatment was evaluated in a group of patients diagnosed with mild asthma who had at least one allergic sensitivity (mainly dust mites, cat epithelium, and grasses). A weekly dose of 0.5 mg/kg was administered as a 5-min intravenous infusion for 9 weeks. The main result was the decrease in serum IgE levels within the first doses, as well as the decrease in symptoms (shortness of breath, chest tightness, wheezing, cough, and sputum) in relation to the allergen challenge. However, no difference was shown in lung function tests compared to the control group [28]. In that same year, the first study was carried out to evaluate the effectiveness in the management of allergic rhinitis; 240 patients from 7 different centers in the United States were included, all of them with allergic sensitivity to ragweed. They were divided into five groups with different doses and routes of administration over 12 weeks period. Weekly doses were administered in the first 2 weeks and then biweekly, the safety of this treatment was demonstrated in this trial, and no difference was found in pharmacokinetics properties when administered intravenously or subcutaneously [29]. However, these studies did not show significant differences in control of asthma or rhinitis symptoms compared to control groups. It was until 1999 that the first trial was published in moderate and severe asthma, in patients aged between 11 to 50 years., who required management with inhaled and systemic corticosteroids and in whom allergic sensitivity to at least 2 aeroallergens was proven; the effectiveness of Omalizumab treatment was evaluated in a period of 20 weeks administering a dosage schedule during the first week: on days 0 (half dose), 4 (half dose) and 7 (full dose) with additional biweekly doses, using 2 different treatment schemes: high dose (5.8 mcg/kilogram/ng of Ig/mL) or low dose (2.5 mc/kilogram/ng of IgE/mL), it was reported a 40% difference in symptoms in the low-dose group (*p* = 0.005) and 42% in the high-dose group (*p* = 0.008) compared to control group, also the need for systemic corticosteroids was reduced by 50% and 65% in high-dose group (*p* = 0.045) and low-dose group (*p* = 0.11), respectively; Moreover, a single dose of Omalizumab rapidly reduced serum free IgE serum concentrations by more than 95% [27].

The first trial conducted in Europe (Sweden, Finland, and Norway) was published in 2000. In this study, 251 patients diagnosed with allergic rhinitis sensitive to birch pollen were divided into two treatment groups. In the first one, patients with serum IgE levels less than 150 IU/mL received 300 mg of subcutaneous Omalizumab at weeks 0 and 4 while the second group with serum IgE levels above 150 IU/mL also received 300 mg of subcutaneous Omalizumab over 6 weeks period a (weeks 0, 3 and 6). The safety of this treatment was demonstrated, as well as a decrease in the need for treatment of antihistamine rescue. Moreover, patients having Omalizumab showed a statistically significant difference in symptoms as well as in the quality of life compared to the control group [30]. Similarly, asthmatic children (334 children aged 6 and 12 years) treated with omalizumab significantly reduced the use of a daily dose of inhaled corticosteroids (ICS), and the withdrawal of this medication was greater in those with Omalizumab compared with placebo (55% vs. 39%, respectively; *p* = 0.004) [21]. Serum IgE levels ranged from 30 to 1300 IU/mL; however, following treatment, levels of this immunoglobulin decreased by 95–99% on average [31]. Omalizumab dose was given at 0.016 mg/kg/IgE [IU/mL] per 4 weeks. 

The first systematic review by the Cochrane collaboration was published in 2004, which included the results of 8 studies and 2037 patients with a diagnosis of moderate to severe asthma, where a decrease in the need for systemic corticosteroid uses by 50% (OR 2.44, 95% CI 1.93–3.08). Likewise, a lower probability of presenting asthma exacerbation was demonstrated in patients treated with omalizumab (OR 0.47, 95% CI 0.37–0.6) [32].

Omalizumab in other diseases. Omalizumab has proved useful in chronic urticaria. The first series of 3 patients diagnosed with chronic urticaria, with variable levels of serum IgE, who did not improve with conventional treatment, was published in 2007. Chronic spontaneous urticaria (CSU) is defined as the development of wheals, angioedema, or both that last for at least 6 weeks. 50% of these patients have an autoimmune background with either an anti-IgE or anti-IgE receptor triggering the release of histamine; Theoretically, the mechanism by which patients with chronic urticaria can benefit from Omalizumab is due to the decrease in the expression of specific receptors for IgE, so anti-IgE and anti-IgE receptor antibodies cannot trigger their function [33]. The first meta-analysis of randomized clinical trials comparing using omalizumab versus placebo in the management of chronic urticaria showed that patients treated with omalizumab had remission rates, defined as a UAS (urticaria activity score 7) of 0, up to 36% after treatment with doses of 300 mg subcutaneous every 4 weeks for 24 weeks. Likewise, it was shown that there is a safety profile similar to that found in a placebo. (RR, 6.55; 95% CI, 4.17–10.28; *p* < 0.00001) [34]. In the most recent systematic review, it is shown that using omalizumab at a dose of 300 mg every 4 weeks is related to an improvement in quality of life, evaluated by the result obtained by the chronic urticaria quality of life questionnaire. (Mean difference −4.03; 95% CI −5.56 to −2.25) [35].

The administration of omalizumab before the start of allergen-specific immunotherapy in patients with allergic asthma, in whom symptoms persisted after administering optimal doses of inhaled steroid, showed a decrease in allergic reactions induced by allergen-specific immunotherapy, compared with placebo (13.5% vs. 26.2; *p* = 0.017; 95% CI 2.91–22.56%); Likewise, using Omalizumab increased the rate of patients who reached maintenance dose (87.3% vs. 72.1%, respectively; *p* = 0.004) [36].

**Figure 2:**
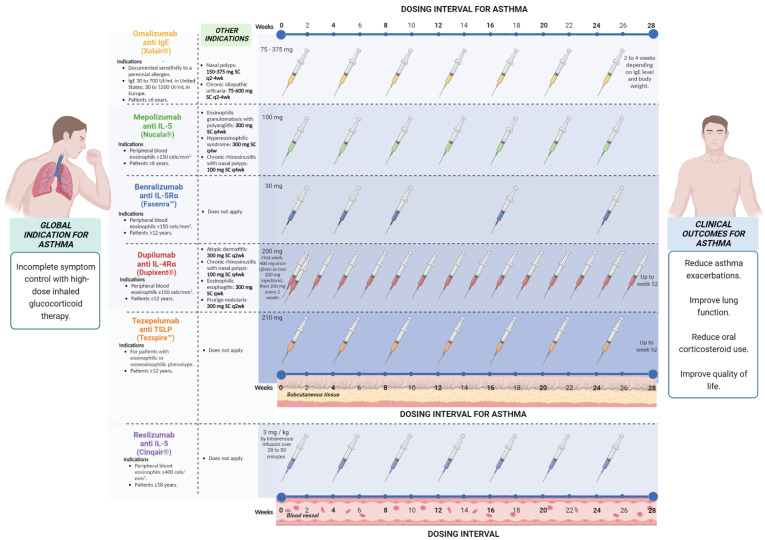
Doses and eligibility criteria of targeted therapy that has been approved for asthma.

Doses and eligibility criteria (Figure 2). The safety and efficacy of Omalizumab have been evaluated, and it has been approved for use in individuals over 6 years of age with a diagnosis of moderate to severe allergic asthma and in whom allergic sensitivity has been demonstrated by an objective method: these patients did not achieve control of their symptoms after management with high-dose of inhaled corticosteroids (ICS) and serum IgE levels between 30–700 IU/mL (in patients older than 12 years) or between 30–1300 IU/mL (in patients 6 to 11 years old) [37]. According to the guidelines published by the global initiative for asthma, the eligibility criteria for this type of therapy in asthma are allergic sensitization (determined by skin prick test or by specific IgE), total serum IgE, and weight within the dose range, and asthma exacerbations during the past year. Likewise, the predictors of good response are serum eosinophils > 260/μL, fraction exhaled nitric oxide (FeNo) > 20 ppb, allergen-driven symptoms, and childhood-onset asthma [38]. The dose of Omalizumab is determined by the serum levels of IgE before biological treatment as well as the weight of each patient, calculated based on 0.016 mg/kg per IU of IgE, with doses for patients older than 12 years between 150–375 mg, subcutaneously every 2–4 weeks, and between 75 and 375 mg every 2 to 4 weeks in patients between 6 and 12 years of age [39]. 

## 3. Mepolizumab

Mepolizumab (Nucala^®^ before called SB-240563) is an N-glycosylated IgG1 (k) antibody that binds to human IL-5 preventing its interaction with the α chain of the IL-5 receptor complex expressed on the cell surface of eosinophils. This cytokine is responsible for the growth, differentiation, activation, recruitment, and survival of eosinophils [40].

Mepolizumab in asthma. The phase 2 trials of Mepolizumab were conducted in early 2002 as a potential treatment for asthma and atopic dermatitis [41]. While in 2000, Leckie et al. demonstrated that a single intravenous infusion of Mepolizumab in patients with allergic asthma reduced the number of circulating blood eosinophils, with suppression maintained over 16 weeks, with no significant effect on the late asthmatic response to allergen challenge or on airway hyperresponsiveness to inhaled histamine [42]. Subsequently, it was demonstrated that Mepolizumab suppresses eosinophils in blood and sputum but did not affect T-cell activation [43]. A significant reduction in bronchial and bone marrow eosinophil counts was seen in patients with mild atopic asthma after three doses of Mepolizumab IV. Additional changes seen in this study include a significant reduction in tenascin thickness, luminal density, procollagen III density, and bronchial mucosal eosinophils expressing Transforming growth factor-beta 1 (TGF-β1) mRNA [44,45,46].

In 2007, Flood-Page et al. reported the use of three doses of Mepolizumab in subjects with moderate to severe asthma with persistent symptoms despite treatment with ICS reaching a 50% reduction in the exacerbation rates but without significance [47]. It was until 2009 that Haldar et al. associated Mepolizumab with significantly fewer exacerbations of refractory eosinophilic asthma (RR: 0.57; 95% CI, 0.32–0.92; *p* = 0.02) and increased asthma-related quality of life in these patients (mean 0.35%; 95% CI, 0.08–0.52; *p* = 0.02), by suppressing eosinophilic airway inflammation, suggested a causal link with the exacerbations. Nevertheless, there was no significant difference in lung function, asthma symptoms, and FeNO [48]. Nair et al. demonstrated a significant reduction in prednisone use without a clinical exacerbation and a decrease in eosinophil number [49]. In DREAM (Dose Ranging Efficacy and Safety with Mepolizumab) study in severe asthma reported that the use of anti-IL-5 represented an important treatment option in patients with severe eosinophilic asthma [50]. Ortega et al. performed a cluster analysis to identify distinctive characteristics within subgroups of patients included in the DREAM trial. Higher decrease in exacerbations was observed in the patients treated with Mepolizumab and elevated eosinophils. The low airway reversibility was associated with a reduction in exacerbations by 53% in the non-obese compared with obese patients [51]. The route of administration was suggested to influence the response to Mepolizumab. The double-blind study, Mepolizumab as adjunctive therapy in patients with severe asthma (MENSA), demonstrated a reduction by 47% of the exacerbations rate in intravenous administration and by 53% in patients receiving subcutaneous SC, both being significantly (*p* < 0.001) [52]. In another study, Ortega et al. demonstrated that Mepolizumab produced a clinical reduction in the exacerbation rate and an improvement in asthma control and quality of life (measured by SGRQ) in patients with severe eosinophilic asthma who had at least two exacerbations in the previous year and had a baseline blood eosinophil threshold of a minimum of 150 cells/mm^3^ [53]. In a randomized, double-blind, placebo-controlled, parallel-group, multicenter, phase 3b trial (MUSCA), Mepolizumab was associated with an important improvement in health-related quality of life, asthma control, lung function, and asthma symptoms [54]. Mepolizumab demonstrated comparable efficacy with Benralizumab, reducing the exacerbations and improving pre-bronchodilator forced expiratory volume in 1 s (FEV_1_) [55].

In 2018, Henriksen et al., in a systematic review and meta-analysis, reported a significant clinical improvement in exacerbation rate and oral corticosteroids (OCS) reduction with Mepolizumab and Reslizumab equally, besides improvement in FEV_1_, asthma control and asthma-related quality of life, with no differences between both drugs in efficacy and safety measures [56]. Kelly E. et al. described that the exacerbation events in Mepolizumab treatment were less severe in terms of symptoms and less responsive to prednisolone, associated with a lower induced sputum eosinophil count compared with placebo [57]. In a multicenter, open-label, long-term safety study (COLUMBIA), patients who participated in the DREAM study with severe asthma reported injection-site reactions and headaches [58]. In 2019 Carpagnano et al. reported the efficacy of Mepolizumab in patients with severe uncontrolled asthma and diagnosed bronchiectasis, suggesting that the presence of these could be a criterion to identify an emerging phenotype of severe eosinophil asthma [59]. Claude S. et al. showed the ability of Mepolizumab to improve small airway function, associated with clinical control of the disease [60].

Mepolizumab in other diseases. Mepolizumab function by blocking IL-5 and makes it a candidate to treat various eosinophil-associated disorders. Most investigated are CRSwNP, eosinophilic esophagitis, hypereosinophilic syndrome (HES), eosinophilic granulomatosis with polyangiitis, allergic bronchopulmonary aspergillosis and eosinophilic chronic obstructive pulmonary disease. In 2004, a study demonstrated that anti-IL5 appeared to be safe in 4 patients with manifestations of hypereosinophilic syndromes, suggesting that Mepolizumab may exhibit a steroid-sparing effect in the disease [61]. Consistent with this observation, Roufosse et al. demonstrated that 750 mg of once a month of Mepolizumab produced satisfactory results in HES patients [62]. In phase 3, a randomized, placebo-controlled trial, it was further demonstrated a 50% reduction in HES symptoms during 32 weeks of treatment compared with a placebo [63]. Interestingly, patients treated with 750 mg of Mepolizumab monthly who were receiving topical corticosteroid therapy and had severe, recurrent bilateral nasal polyps showed a significant improvement in efficacy outcomes after 9 weeks of treatment compared with placebo-treated patients [64]. Cavaliere et al. presented a case report associated with an improvement in olfactory capacity after 4 months of treatment [65]. About eosinophilic esophagitis (EoE) is an eosinophilic inflammation of the esophagus. The first trial with Mepolizumab treatment was in 2010, with a significant reduction in esophageal eosinophilia after 4 weeks of Mepolizumab treatment; also reduced the expression of TGF-β1 and tenascin C (TN-C) in the esophageal epithelium after 13 weeks of treatment [66]. However, a systematic review and network meta-analysis that compared the efficacy of six pharmacologic treatments, budesonide suspension, and couscous, fluticasone, esomeprazole, prednisone, Mepolizumab, and placebo, found viscous budesonide, to be the most effective therapy for EoE [67].

Doses and eligibility criteria (Figure 2). According to Global Strategy for Asthma Management and Prevention (GINA) 2022, the doses approved for patients with 12 years and older are 100 mg SC every 4 weeks, and for patients with 6 to 11 years, a dose of 40 mg SC every 4 weeks, both with a minimum of 4 months of treatment. The eligibility criteria vary but usually include the number of severe exacerbations in the last year and blood eosinophils (>150 or >300/m) with a different cut point for patients with OCS. Potential predictors of good response the higher blood eosinophils, higher number of severe exacerbations in previous years, adult-onset asthma, nasal polyposis, maintenance of OCSs at baseline, and low lung function (FEV_1_ < 65% predicted in one study) [38].

## 4. Reslizumab

Reslizumab (Cinqair^®^, before SCH55700) is an IgG4/k humanized monoclonal antibody composed of the complementarity-determining regions of a murine antibody to human IL-5. It neutralizes circulating IL-5 by preventing its binding to eosinophils [68].

Reslizumab for asthma. Pre-clinical trials showed that reslizumab inhibited both pulmonary eosinophilia and airway hyperresponsiveness [69]. The first clinical trial was developed in 2003 that showed a decreased blood eosinophils dose-dependently, with a single dose of 1.0 mg/kg—the decrease remained significant up to day 30; a trend toward improvement in baseline FEV_1_ was observed too, but no significant changes occurred in other clinical indices of disease activity [69]. In 2011, Castro et al. conducted the first controlled clinical trial in which they identified a phenotype of patients with asthma who benefited from anti–IL-5 therapy. They documented a reduction in eosinophils, and an improved level of asthma control and airway function, particularly in patients with higher ACQ scores and nasal polyps [70]. 

The same group undertook two clinical trials in phase 3 and reported a significant frequency reduction in asthma exacerbations in patients with asthma and blood counts of eosinophil > 400 cells/µL that receive intravenous Reslizumab (3.0 mg/kg) every 4 weeks for 1 year (RR, 0.50; 95% CI: 0.37–0.67) and (RR, 0.41; 95% CI: 0.28–0.59) in each study (both *p* < 0.0001) compared with those receiving placebo, these studies could show significant changes in FEV_1_, Asthma Quality of Life Questionnaire (AQLQ), Asthma Control Questionnaire-7 (ACQ-7), Asthma Symptom Utility Index (ASUI) scores, and blood eosinophil count at 16 and 52 weeks of treatment [71]. One year later, Corren et al. reported the results of another randomized, double-blind, placebo-controlled, phase 3 trial in the United States. They conducted the study in a population with asthma unselected for baseline blood eosinophil counts to determine the impact of baseline eosinophil level on efficacy. It confirmed the benefit lung function of intravenous Reslizumab (3.0 mg/kg) every 4 weeks only in patients with asthma and blood eosinophil counts >400 cells/mL; the subgroup with eosinophils > 400 cells/mL, the treatment with Reslizumab was associated with a significant increase in FEV_1_ (0.270 + 0.1320 L, *p*: 0.0436) compared to the subgroup with eosinophils < 400 (0.033 + 0.0539 L, *p*: 0.5422), this trial didn’t report a significant difference in ACQ-7 and SABA use in both subgroups [72]. An open-label extension study of these phase III trials was reported in 2017. Long-term efficacy and safety were evaluated in moderate-to-severe eosinophilic asthma. Patients received Reslizumab 3.0 mg/kg intravenously every 4 weeks for up to 24 months, patients that previously received placebo were enrolled to receive Reslizumab, FEV_1_, and ACQ score had a significant change at 4 and 16 weeks of treatment. However, blood eosinophil counts appeared to be returning to baseline after Reslizumab discontinuation. The most common adverse effect reported was worsening asthma (29%), nasopharyngitis (14%), and respiratory tract infections (10%). There were two drug hypersensitivity reactions reported and two events of drug eruption reported; all were related to other allergen exposure and did not result in discontinuation of Reslizumab, and only 2% discontinued treatment because of adverse events [73].

Doses and eligibility criteria (Figure 2). Reslizumab is indicated in patients aged >18 years with severe eosinophilic asthma uncontrolled dose approved is 3 mg/kg by intravenous infusion every 4 weeks; potential predictors of good response are the same as those recommended for Mepolizumab and Benralizumab [38].

## 5. Benralizumab

Benralizumab (FasenraTM before called MEDI-563) is a humanized fucosylated Mab recombinant immunoglobulin IgG1K isotype, which binds to the IL-5 with high affinity to the domain 1 of the α-chain of the IL-5 receptor [74,75]. A fucosylation of the oligosaccharide of IgG1 results in an up to 50 times higher affinity to Fcg receptor (FcgR) expressed on natural killer cells, macrophages, and neutrophils [76]. Fucosylation of the Benralizumab enhances the interaction more than 1000 times. Therefore, it can increase the functions of antibody-dependent cell-mediated cytotoxicity [75]. These characteristics may result in a complete depletion of eosinophils in the airway lumen without degranulation of eosinophils [77].

Benralizumab in asthma. It was first described in knockout mice in 2009 by Koike et al., as a strong hIL-5Ralpha neutralizing mAb hIL-5Ralpha that blocks the binding of the IL-5 ligand to its receptor [74]. Evidence for its role in asthma derived from a study showing patients that mild atopic asthmatics giving a single dose from 0.03 mg/kg to 3 mg/kg depleted circulating eosinophils at 24 h, and the effect persisted for at least 2 to 3 months [78]. Laviolette et al. conducted another phase 1 study. It was a multicentered, randomized, double-blinded, and placebo-controlled study from 2008 to 2011 in asthmatics that had a sputum eosinophil count ≥2.5%. In this study, there was a reduction in 100% of eosinophils in both sputum and blood in the group of asthmatic patients treated with Benralizumab (1 mg/kg single IV or SC) per day 28 [79].

In a separate study, benralizumab was applied at three different doses (2 mg, 20, and 100 mg) to patients with eosinophilic asthma who were using medium-dose or high-dose inhaled corticosteroids and long-acting β agonists. Interestingly, those patients with benralizumab doses of 20 mg and 100 mg showed lower exacerbations than the placebo group proving its usefulness in adults with uncontrolled eosinophilic asthma [80]. Similar outcomes were presented by Nowak et al., who reported a 49% reduction in exacerbations and hospitalizations rates [81]. The first phase 3a studies, SIROCCO AND CALIMA, showed that Benralizumab given as a 30 mg dose for 4 to 8 weeks for 48 weeks (the first three doses were given every 4 weeks) resulted in improved lung function and fewer exacerbations rates in the 8-week cohort [82,83]. In this phase 3, studies researchers concluded that the effects seemed to be better in patients with blood eosinophilia >300 cells/µL [77]. In contrast, low to medium doses of ICS or low dosage and LABA did not improve significantly in subjects with- or without eosinophilia [73). The first phase 3a studies, SIROCCO AND CALIMA, showed that Benralizumab given as a 30 mg dose every 4 to 8 weeks for 48 weeks (the first three doses were given every 4 weeks) resulted in improved lung function and fewer exacerbations rates in the 8-week cohort [82,83]. These phase 3 studies observed that the therapeutic effects seemed to be better in patients with blood eosinophilia >300 cells/µL [77]. Belralizumab also plays an important role in reducing the use of corticosteroids (OCS). Indeed [73,74,75,76], the phase 3a study called ZONDA showed that Benralizumab at 30 mg dose given every 4 to 8 weeks reduced the use of OCS by 75% as compared with a reduction in 25% in the OCS doses in the placebo group [84]. As an add, Blecker and Nair found an improvement in the quality of life assessed with the Asthma Quality of Life Questionnaire for 12 years and older (AQLQ(S) + 12 scores; 0.30, 0.10–0.50; *p* = 0.0036), (95% CI, 0.14 to 0.76 *p* = 0.004) [82,83,84].

The US Food and Drug Administration (FDA)’s approved Benralizumab in 2017 for the add-on maintenance treatment of patients with severe asthma aged 12 years and older and with an eosinophilic phenotype [77,85]. In 2019, Liu et al. published a meta-analysis about the adverse events of Benralizumab in moderate to severe eosinophilic asthma considering previous studies which described the risk of headache (RR 1.42, 95% CI 1.07–1.87) and pyrexia (RR 2.26, 95% CI 1.32–3.87) was higher in the Benralizumab group. However, there was a reduced risk of serious adverse effects and decreased moderately to severe asthma symptoms [86]. In 2019, the GRECO study reported that Benralizumab was safe for patients who were under treatment administered by themselves or a caregiver at home. Despite this, it was recommended that the first three doses should be applied by the physician’s office or with supervision. Only 1.7% of autoinjector administrations were unsuccessful during 28 weeks of treatment (98.3% successful administration; 95% CI: 91.41–99.05) [87]. BORA trial was a 2-year follow-up of the patients enrolled in the SIROCCO and CALIMA studies. It reported no new consequences of long-term eosinophil depletion. The exacerbation rate was (0.48; 95% CI 0.42 to 0.56) and (0.46; 95% CI 0.39 to 0.53) for the 4 and 8 weeks groups, respectively [88]; following the same trend, Korn et al. published in 2021 a 5 year follow up of 345 subjects who were in the SIROCCO, CALIMA, and ZONDA trials, this integrated analysis reported that the adverse events and serious adverse events were stable over the 5 years and did not increase with higher Benralizumab exposure [89].

Benralizumab in other diseases. In recent years, Benralizumab has been used to treat other diseases, such as chronic obstructive pulmonary disease (COPD), and to date, three-phase trials have investigated the impact of Benralizumab on COPD exacerbation rates. Unfortunately, there is no evidence that this treatment can ameliorate exacerbation outcomes for most patients with COPD [90]. In contrast, 30% of patients with polyps treated with Benralizumab no longer required surgery at week 25. Moreover, there was a significant improvement in nasal polyposis severity, visual analog scale score, and endoscopic nasal polyp score [83]. There is not enough evidence that supports Benralizumab as a treatment in patients with no response to antihistamines with chronic spontaneous urticaria. The role of Benralizumab was studied in a small group of patients (*n* = 12) suffering from chronic spontaneous urticaria who were unresponsive to second-generation H1-antihistamines in a 24-week, single-blind study. At the completion of the treatment, symptoms decreased by −15.7 points in the urticaria activity score during a 7-day interval (95% CI, −6.6 to −24.8; *p* < 0.001); only nine patients completed the study, five patients had a very good response, two patients had only a partial response, the rest of the patients showed no improvement. However, a trial in a larger patient population is required [91].

Doses and eligibility criteria (Figure 2). The FDA has approved Benralizumab for the add-on maintenance treatment of patients with severe asthma aged 12 years and older and with an eosinophilic phenotype. It is not indicated for the treatment of any other eosinophilic conditions, including acute bronchospasm relief or status asthmaticus [75]. Step 5 of the GINA main report is established to add-on anti-interleukin/5R treatment (subcutaneous Benralizumab for ages ≥12 years) with severe eosinophilic asthma that is uncontrolled in Step 4 [38]. The recommended dose is 30 mg, administered once every 4 weeks for the first three doses and then once every 8 weeks thereafter by subcutaneous injection into the upper arm, thigh, or abdomen. 

## 6. Dupilumab

Dupilumab (Dupixent^®^ previously called REGN668) is a recombinant IgG4 monoclonal antibody against the IL-4/IL-13 alpha receptor, avoiding the subsequent activation of type-2 inflammation pathways [92,93]. Dupilumab also reduced the expression of genes involved in type 2 inflammation (IL13, IL31, CCL17, CCL18, and CCL26), epidermal hyperplasia (keratin 16 and MKi67), T cells, dendritic cells (ICOS, CD11c, and CTLA4), and TH17/TH22 activity (IL17A, IL-22, and S100As) and concurrently increased expression of epidermal differentiation, barrier, and lipid metabolism genes (filaggrin, loricrin, claudins, and ELOVL3). Additionally, this biological agent suppressed type 2 CCL17, CCL18, periostin, and total and allergen-specific IgE. [94].

Dupilumab in asthma. Several clinical trials have evaluated the efficacy of the anti-IL-4 receptor in asthma at different doses and times (EXPEDITION, P2b, QUEST, VENTURE, and TRANSVERSE). Most clinically evaluated tests include changes in lung function (FEV1), the score of specific quality life questionnaires (ACQ-5 and AQLQ), and/or levels in biomarkers (serum eosinophils and IgE levels) [95]. 200–300 mg of Dupilumab every 2 to 4 weeks were used in most clinical trials [95]. For example, the LIBERTY study evaluated the administration of 200 and 300 mg of Dupilumab applied every 15 days for one year; both dosages reduced the annualized rate of asthma exacerbation and improved around 300 mL of the FEV_1_ at week 12 when were compared with the placebo group; these results were maintained even when stratified by eosinophil levels. Likewise, asthma control was better with Dupilumab from the second week, an effect that persisted throughout the entire study [96].

Dupilumab has also been evaluated in 6 to 11 children in a multicenter study with severe asthma. Doses of 100 mg were given if the patient weighed less than 30 kg and 200 mg if heavier, every two weeks for 52 weeks); Dupilumab reduced asthma exacerbations at one year, improved 10% the pre-bronchodilator FEV_1_, and reduced inflammatory biomarkers such as Thymus and activation-regulated chemokine (TARC), FENO, and total IgE compared to the placebo group. In addition, patients with anti-IL-4 therapy improved asthma control at 24 weeks. Regarding safety in this group, approximately 10% of patients developed viral upper respiratory tract infections, which was the main adverse reaction [97].

Dupilumab in other diseases. Dupilumab has also been used in moderate-to-severe atopic dermatitis. The SOLO1 and SOLO2 studies enrolled adults from the USA, Europe, and Asia with moderate-to-severe atopic dermatitis whose disease was inadequately controlled by topical steroids. They applied 300 mg of Dupilumab weekly or every two weeks for 16 weeks, and the therapeutic targets were compared versus the placebo group. Both groups showed a reduction in clinical symptoms at 16 weeks, as the extension and severity of atopic eczema by EASI score decreased the pruritus around 3 points of peak score, being these changes notorious from week 2. Patients also reported improvements in pruritus, sleep, symptoms of anxiety or depression, and quality of life. The placebo group had more need for rescue therapy for control of symptoms. Reactions at the injection site and conjunctivitis were more frequent in the Dupilumab groups than in the placebo [98]. Similar results were reported in the LIBERTY AD CHRONOS trial (NCT02260986). This analysis compared dupilumab (300 mg every 2 weeks for 1 year) plus topical steroid versus topical steroid alone. The Dupilumab group had a lower annualized flare rate (78% reduction in annual flare-ups) than the topical steroid group suggesting that Dupilumab can be used as therapy in adults with moderate to severe AD by providing continuous, long-term disease control [99]. To investigate mechanisms mediating the effects of Dupilumab, a transcriptomics analysis of skin biopsies was carried out in atopic dermatitis patients administered 200 mg weakly. Interestingly, Dupilumab significantly reduced the expression of genes associated with Th 2 inflammation (IL13, CCL17, CCL18, CCL26, and IL31), epidermal hyperplasia (keratin 16 and MKi67), T cells, and dendritic cells while increased expression of epidermal differentiation, barrier, and lipid metabolism genes (filaggrin, loricrin, claudins, and ELOVL3). Dupilumab also reduced cellular infiltrates, including CD31 T cells. The changes in gene expression were observed at 4 weeks and even higher at week 16. In contrast, there was no comparable modulation in placebo-treated patients [94].

Patients suffering from CRSwNP require recurring systemic corticosteroid and sinus surgery repeatedly. Two multinational, multicentre, randomized, double-blind, placebo-controlled studies, the LIBERTY NP SINUS-24 and the LIBERTY NP SINUS-52, assessed Dupilumab in adults with severe CRSwNP [93,94]. Dupilumab was given at 200 mg every two weeks for 24 and 52 weeks, diminished nasal congestion and nasal polyp score from week 4 and still improved at week 24. Other clinical and radiographic targets also showed improvement as the Lund MacKay score, or SNOT-22 score [100]. Similar findings have been reported in a study using 300 mg of Dupilumab weekly. There were improved clinical-radiographic outcomes after 16 weeks of treatment in comparison to the placebo. Decreased Th2 inflammation biomarkers were also observed in both nasal secretions and nasal tissue [101].

In the surgical scenario, Dupilumab demonstrated significant improvements in disease signs and symptoms and reduced need for both sinonasal surgery and the use of systemic corticosteroid compared to placebo in patients with severe nasal polyposis, regardless of the use of SCS in the previous 2 years, or previous sinonasal surgery [102]. These findings support the use of adding Dupilumab in patients with severe CRwNP with asthma [103].

The contraindications for the use of Dulipumab include another chronic lung disease, pregnancy, breastfeeding, active parasitic infection, HIV infection or immunosuppression condition, and eosinophils count >1500 cell/mm^3^ [104].

Doses and eligibility criteria (Figure 2). Dulipumab is approved in the USA and Europe for treating moderate-to-severe asthma, CRSwNP, and atopic dermatitis [92]. For its prescription in asthma, patients must have ICS at medium or high doses in addition to OCS [104] According to GINA, patients with severe eosinophilic/type 2 asthma are eligible when they have a history of exacerbations in the last year and blood eosinophils > 150 and <1500 cells/µL or FeNO > 25 ppb or taking maintenance oral corticoids. Children 6–11 require a weight-dependent dose and frequency by subcutaneous injection for age >12 years, 200 or 300 mg by subcutaneous injection every 2 weeks for patients with OCSs-dependent severe asthma or concomitant moderate/severe atopic dermatitis, 300 mg by subcutaneous injection every 2 weeks. Patients with chronic rhinosinusitis with polyps should be considered due to the benefits (reduced size of nasal polyps, improved nasal symptoms, and reduced need for OCSs or surgery).

## 7. Tezepelumab

Tezepelumab (TezspireTM before called AMG157/MEDI9929) is a human anti-TSLP monoclonal immunoglobulin G2λ that binds human TSLP and prevents its interaction with receptor complex. TSLP is an epithelial-cell–derived cytokine that is produced in response to proinflammatory stimuli and drives allergic inflammatory responses through its activity on innate immune cells, including dendritic cells, mast cells, and CD34+ progenitor cells [105,106].

Tezepelumab in asthma. A proof-of-concept, randomized, double-blind, placebo-controlled study demonstrated that Tezepelumab attenuated the early- and late asthmatic responses to allergen challenges in mild allergic asthma patients. In this study, 700 mg of Tezepelumab was given in three monthly doses or placebo intravenously, and allergen challenges were carried out on days 42 and 84 to evaluate the late asthmatic response, as measured 3 to 7 h after the allergen challenge. Indeed, Tezepelumab prevented allergen-induced bronchoconstriction by 34.0% and 45.9% on days 42 and 84, respectively, as determined by the FEV_1_ measurements in asthmatics compared with the normal group. In addition, levels of blood- and sputum eosinophils decreased before and after the allergen challenge and in the fraction of exhaled nitric oxide [106].

Additional studies have evaluated the therapeutic effects of Tezepelumab. Corren et al. conducted the phase 2b study PATHWAY, a randomized, placebo-controlled, dose-ranging trial with subcutaneous Tezepelumab in adults with moderate to severe, uncontrolled asthma and a history of exacerbations during the year, and they randomized patients for received low dose (70 mg every 4 weeks), medium dose (210 mg every 4 weeks) or high dose (280 mg every 2 weeks) or of placebo every 2 weeks over 52 weeks. Prebronchodilator FEV_1_ was higher in all Tezepelumab groups than in the placebo group; In addition, a reduction in annualized rate of asthma exacerbations was reported: 62% (90% CI: 42–75), 71% (90% CI: 54–82), and 66% (90% CI: 47–79), in low, medium, or high dose Tezepelumab groups, respectively, when was compared to placebo (*p* < 0.001) [107]. A post hoc analysis of data from the PATHWAY phase 2b study reported that treatment with Tezepelumab resulted in clinically meaningful improvements in asthma control; the proportion of ACQ-6 responders (reduction in >0.5 in ACQ-6 total score) and of AQLQ(S)+ 2 responders (increase in overall AQLQ(S) + 12 scores of >0.5), 82% and 77% of tezepelumab-treated patients, respectively, were higher at week 50 compared with 70% and 64% of placebo-treated patients, respectively [108].

Two phase 3, multicentre, randomized, double-blind, placebo-controlled, parallel-group studies were conducted to evaluate the efficacy and safety (NAVIGATOR and SOURCE) of Tezepelumab treatment in patients with severe asthma. NAVIGATOR trial was made in adults and adolescents with uncontrolled asthma with medium- or high-dose inhaled glucocorticoids and at least additional asthma controller medication with or without oral glucocorticoids. Patients received 210 mg of Tezepelumab or placebo subcutaneously every 4 weeks for 52 weeks. Consistent with what has been reported, a reduction in the annualized rate of asthma exacerbations was showed: 0.93 (95% CI: 0.80–1.07) in Tezepelumab-treated patients vs. 2.10 (95% CI: 1.84–2.39) in placebo-treated patients (rate ratio, 0.44; 95% CI, 0.37 to 0.53; *p* < 0.001); in addition, prebronchodilator FEV_1_ and ACQ-6, AQLQ + 12, and ASD scores were better in Tezepelumab group [109].

The SOURCE trial was conducted in adults with oral corticoid-dependent asthma to evaluate the effect of Tezepelumab 210 mg administered subcutaneously every 4 weeks on oral corticoid dose reduction; eligible patients must have been receiving a stable dose of 7.5–30 mg prednisone for >1 month. Authors did not observe a significant improvement in OCS dose reduction with Tezepelumab versus placebo in the overall population at week 48; however, when the analysis was performed on subgroups categorized by blood eosinophil counts, Tezepelumab increased the cumulative odds of achieving a category of greater percentage reduction (although not significantly) in daily maintenance oral corticoids (OR, 2.58; 95% CI: 1.16–5.75 and OR, 3.49; 95% CI: 1.16–10.49) in patients with ≥150 cells/μL or ≥300 cells/μL, respectively, compared with placebo [110].

DESTINATION is an ongoing Phase 3 randomized, double-blind, placebo-controlled trial to assess the long-term safety and tolerability of Tezepelumab over 104 weeks that it is ongoing. The study population comprised patients who completed the 52- and 48-week NAVIGATOR and SOURCE studies, respectively [111].

PATH-HOME is a phase 3, multicenter, randomized, open-label, parallel-group study that enrolled adults and adolescents with severe, uncontrolled asthma to assess the functionality and performance of an accessorized pre-filled syringe (APFS) and an autoinjector (AI) for the administration of Tezepelumab in the clinic and at home. Both devices were effectively employed. Tezepelumab administered via APFS was successful by 91.7% of the participants and via AI by 92.4%. After 24 weeks of treatment, improvements in ACQ-6 score were recorded in 81.1% and 76.2% of the patients in the APFS and AI groups, respectively. Nasopharyngitis was reported as the most common adverse effect (9.3%). Injection-site reactions occurred in 5.7% (AI) and 0% (AFPS) of the patients [112]. Taken together, these results from phase 2/3 trials suggest that blocking the upstream alarmin TSLP with tezepelumab results in clinically meaningful improvements in asthma control in patients with T2-high asthma concerning exacerbations. 

Tezepelumab and another indication. ALLEVIAD is a phase 2a, randomized, double-blind, placebo-controlled study that assesses the effect of subcutaneous Tezepelumab 280 mg or placebo every 2 weeks, plus class 3 topical corticosteroids in severe atopic dermatitis. The primary endpoint was the response rate for a >50% reduction in the Eczema Area and Severity Index (EASI50) at week 12. The treatment difference was not statistically significant (OR, 1.97; 95% IC: 0.90–4.33; *p* = 0.091). However, there was a numerically greater percentage of tezepelumab-treated patients who achieved EASI50 (64.7%) versus placebo-treated (48.2%) [105].

Doses and eligibility criteria (Figure 2). According to the GINA report 2022, the dose approved for Tezepelumab is 210 mg subcutaneously every 4 weeks for patients aged > 12 years with severe asthma. Greater benefits (reduction in severe exacerbations) should be considered in patients with high blood eosinophil or high FeNO. There are no recommendations of benefit in patients with severe asthma who have no evidence of Type 2 inflammation [38]. A systematic review of the EAACI guidelines on the use of biologicals in severe asthma in 2020 evaluated Benralizumab, Dupilumab, Mepolizumab, Omalizumab, and Reslizumab and mentioned a high reduction in severe asthma exacerbations as add-on therapy for patients with severe uncontrolled eosinophilic asthma. Regarding the reduction in the daily dose of OCS, only Benralizumab, Dupilumab, and Mepolizumab showed a reduction compared with the standard of care [113].

## 8. New Monoclonal Antibodies in Asthma and Other Diseases with Type 2 Inflammation

Itepekimab: IL-33 induces the response of the Th2 adaptive immunity through a complex including the binding of ST2 protein, which is in the membrane of mast cells, basophils, eosinophils, T cells, dendritic cells, ILC2 and NK cells [114]. In vivo trials demonstrated that IL-33 stimulates Th2 cytokine expression, with increased eosinophils levels due to an increase in IL-5 expression and IgE synthesis by IL-4 stimulation, identifying that IL-33 induces gene expression of IL-13 in all tissues, expression of both IL-4 and IL-5 was higher in thymus, spleen, liver, and lung [115]. Itepekimab is a human IgG4P monoclonal antibody against interleukin-33. According to the current nomenclature, it has the infix -*ki*- to refer to its target class (cytokine). In a phase 2 trial, 296 patients with moderate-to-severe asthma were randomly assigned to receive: Itepekimab monotherapy (300 mg subcutaneous), Itepekimab plus Dupilumab (300 mg subcutaneous of each one), Dupilumab monotherapy (300 mg subcutaneous) or placebo every 2 weeks for 12 weeks. Two efficiency endpoints were analyzed, the primary being loss of asthma control; 22% of patients have a loss of asthma control in the Itepikimab group, 27% in the combination group, 19% in the Dupilumab group, and 41% in the placebo group. The OR were calculated comparing each group versus placebo: Itepekimab monotherapy, 0.42 (95% CI, 0.20 to 0.88; *p* = 0.02); combination therapy, 0.52 (95% CI, 0.26 to 1.06; *p* = 0.07); and dupilumab monotherapy, 0.33 (95% CI, 0.15 to 0.70; *p* value not reported). The secondary endpoint was the change in the prebronchodilator FEV_1_ from baseline to week 12. An increase in this was reported in the groups with monotherapy (Itepekimab or Dupilumab) but not in the combination group when compared to placebo [116].

Brodalumab. IL-25 (IL-17E) is produced by bronchial subepithelial mucosa cells, such as basophils, eosinophils, and mast cells, up-regulated their receptors on basophils, inhibiting their apoptosis and promoting their degranulation [117,118]. It has been demonstrated ex vivo that IL-25 is associated with angiogenesis through its effects on the proliferation of endothelial cells and the expression of vascular endothelial growth factor and with airway remodeling, maybe because fibroblasts and smooths muscle cells also express IL-25R, both factors contributing to asthma severity [119]. IL-25 binds to its receptor composed of IL-17RA and IL-17RB for signal transduction, IL-17RA being the key component used by IL-25. IL-17 family cytokines (IL-17A-17F) play an essential role in various diseases such as autoimmune disorders, of which IL-25 exacerbates allergic inflammation by promoting the production of type 2 cytokines. Brodalumab (AMG 827) is a human anti-IL-17AR immunoglobulin G2 (IgG2) monoclonal antibody that binds to human IL-17RA, blocking IL-25, IL-17A, and IL-17F. In 2013 phase 2a, a randomized, double-blind, placebo-controlled, dose-ranging study evaluated the efficacy and safety of the anti-IL-17 in patients with inadequately controlled moderate to severe asthma, not statistically significant in clinical efficacy on ACQ were observed, neither in lung function nor asthma symptoms [120]. Consequently, further studies are required. 

Tralokinumab and lebrokizumab. IL-13 is produced by Th2 cells, this cytokine is responsible for eosinophils proliferation and macrophage activation and induces globet cell hyperplasia, which increases the production of mucus [121]. Tralokinumab and Lebrokizumab are anti-human IL-13 monoclonal antibodies developed for the treatment of severe, uncontrolled asthma [122,123]. Phase 1 studies have shown that intratracheal administration of human IL-13 specifically inhibited IL-13-induced eosinophil recruitment to the lung and suggested the potential for both anti-inflammatory and possible airway remodeling effects [124,125]. Phase II studies also supported the finding that there was an improvement in FEV_1_ but didn´t show any improvement in quality of life or reduced asthma exacerbation [126,127], but phase 3 studies didn’t show any statistical significance in any of these variables [128,129,130].

Lirentelimab. It is a monoclonal antibody targeting a sialic acid-binding Ig-like lectin 8, an inhibitory receptor that is a receptor expressed on eosinophils and mast cells. In a phase 2 study, Lirentelimab improved symptoms in adults suffering either eosinophilic gastritis or resistant chronic spontaneous and inducible urticaria [131,132].

## 9. Conclusions

Over the last few years, biologicals have been used successfully for the treatment of allergic diseases. They can be effective in reducing the severity and frequency of allergic reactions and may be recommended for individuals who have not responded to other types of treatment. The most successful biologic therapies are those that target cytokines regulating the Th2 response. Surprisingly, targeting chemokines that are involved in the allergic response has not been successful. It has been proposed that the redundancy of the chemokine system and insufficient blockade of the receptors in different cell types may be the cause of the failure to develop an efficient anti-chemokine therapy in allergic diseases. In addition, the demonstration that chemokines are released for a shorter period compared with Th2 cytokines such as IL-5 limits their biological effect at the allergic site. Currently, there are novel biologicals under development. The development of novel monoclonal antibodies targeting the type 2 inflammation pathway increases the potential for success in further improving the treatment of allergic diseases and other conditions related to this type of inflammation.

## Data Availability

Not applicable.
